# A novel insecticide impairs bumblebee memory and sucrose responsiveness across high and low nutrition

**DOI:** 10.1098/rsos.231798

**Published:** 2024-05-08

**Authors:** Lily K. Gray, Marcus Hulsey, Harry Siviter

**Affiliations:** ^1^ Department of Integrative Biology, University of Texas at Austin, Austin, TX 78712, USA; ^2^ University of Oklahoma, Norman, OK 73019, USA; ^3^ School of Biological Sciences, University of Bristol, Bristol BS8 1TQ, UK

**Keywords:** bee behaviour, *Bombus*, interaction effects, pesticide, poor nutrition, pollinators

## Abstract

Wild bees are important pollinators of crops and wildflowers but are exposed to a myriad of different anthropogenic stressors, such as pesticides and poor nutrition, as a consequence of intensive agriculture. These stressors do not act in isolation, but interact, and may exacerbate one another. Here, we assessed whether a field-realistic concentration of flupyradifurone, a novel pesticide that has been labelled as ‘bee safe’ by regulators, influenced bumblebee sucrose responsiveness and long-term memory. In a fully crossed experimental design, we exposed individual bumblebees (*Bombus impatiens*) to flupyradifurone at high (50% (w/w)) or low (15% (w/w)) sucrose concentrations, replicating diets that are either carbohydrate rich or poor, respectively. We found that flupyradifurone impaired sucrose responsiveness and long-term memory at both sucrose concentrations, indicating that better nutrition did not buffer the negative impact of flupyradifurone. We found no individual impact of sugar deficiency on bee behaviour and no significant interactions between pesticide exposure and poor nutrition. Our results add to a growing body of evidence demonstrating that flupyradifurone has significant negative impacts on pollinators, indicating that this pesticide is not ‘bee safe’. This suggests that agrochemical risk assessments are not protecting pollinators from the unintended consequences of pesticide use.

## 1. Introduction

Insect pollination is vital for food security and wild ecosystems. An estimated 87.5% of flowering plants, and 75% of crops, require animal pollination [1,2]. Within insects, wild bees are particularly important pollinators [3–5], but many species are undergoing range declines [6–9]. Wild bee declines are driven by a multitude of different anthropogenic stressors, including pesticide use, and loss of habitat, as a consequence of intensive agriculture [7,10–12]. Pesticide use can have both lethal [13,14] and sub-lethal [15–19] impacts on bees at field-realistic levels, which can influence bee populations [10,11]. Loss of habitat reduces floral resources which can impair bee nutrition [20]. This can alter wild bee communities, and in the worst case, lead to wild bee extirpation [20–22]. While individually, both pesticide exposure and poor nutrition can have a negative influence on bee health, they can also interact. The interactions between these stressors can be: (i) additive, where the two stressors are equal to their combined individual effect; (ii) synergistic, where the two stressors exacerbate one another and have a greater than additive effect; or (iii) antagonistic, where the impact is lower than the predicted additive effect [23]. Previous studies have found that most interactions between pesticides and poor nutrition are additive [23–26] or synergistic [13,14,27–30]. The underlying mechanism that determines the type of interaction (synergistic, additive or antagonistic) between pesticides and poor nutrition is poorly understood and requires further investigation [23].

One potential driver of synergistic interactions between pesticides and poor nutrition impacts on behaviour [23]. Bumblebees forage across a large environment collecting nectar and pollen. Foraging efficiency is important as it increases colony resource intake and reproductive output [31]. Consequently, if anthropogenic stressors influence elements of bee foraging, this may have downstream fitness consequences. Individually, exposure to certain pesticides can impair bee behaviour. For example, exposure to neonicotinoids can reduce bee sucrose sensitivity [32–35], which is directly related to foraging motivation [36]. Pesticides can also influence bee learning and memory across multiple modalities (e.g. olfactory, colour and spatial) [18,37–41], which may have downstream consequences for foraging efficiency [16,42–44]. Once more, exposure to one stressor can influence the behavioural response to another. For example, in a floral environment where nectar concentration is low, bees may visit more flowers to try and meet their nutritional requirements and consequently consume more pesticides [13,29]. By contrast, bees exposed to certain pesticides (e.g. neonicotinoids and sulfoxaflor) respond by reducing nectar consumption that leads to a sugar deficiency [45–48]. However, our understanding of the behavioural response of bees to a combination of pesticides and poor nutrition remains relatively unexplored [23].

Here, in a fully crossed experimental design, we assess the combined and individual impact of (i) the novel insecticide flupyradifurone, and (ii) poor nutrition on bumblebee (*Bombus impatiens*) sucrose sensitivity and long-term memory. Flupyradifurone is a butanolide insecticide and has a similar mode of action to neonicotinoids, targeting nicotinic acetylcholine receptors (nAChRs) [49]. However, owing to differences in their structural activity relationships, flupyradifurone is in a distinct chemical class from neonicotinoids [49]. Flupyradifurone is also systemic and consequently has a number of different applications (e.g. foliar spray, seed treatment or soil drench) and can be present in both the nectar and pollen of treated crops [50]. It is thought by regulators (e.g. Environmental Protection Agency) to pose a low threat to bees and so can be used on bee-visited crops while they are flowering [50]. Despite this, exposure to flupyradifurone at field-realistic concentrations can increase honeybee mortality [51–53] and impair behaviour, influencing learning, memory, thermoregulation, flight and sucrose sensitivity at field-realistic concentrations ([29,51,53,54]; recently reviewed in [55]). Less is known about the impact of flupyradifurone on non-*Apis* bees, but acute exposure to the pesticide can impair bumblebee (*B. impatiens*) learning and memory [38], and chronic exposure can harm bumblebee (*Bombus terrestris*) colony growth [56]. Exposure to Sivanto, a chemical formulation containing flupyradifurone, can also have both lethal and sub-lethal impacts on solitary bees [14,57,58].

In this experiment, we defined poor nutrition as sugar deficiency [59]. Foraging bees collect nectar as their primary source of carbohydrate [60]. Crops and wildflowers range in both the concentration and volume of nectar they contain, and bees aim to maximize their foraging efficiency by visiting the most profitable flowers [61]. However, intensive agriculture reduces floral abundance, and consequently, bees may consume more nectar from a mass flowering crop which contains lower concentrations of nectar [62]. However, even if the volume of nectar consumed by bees increases, the amount of sugar they consume is limited by their capacity to process water [13,63]. This means that even when bumblebees are provided with an ad libitum supply of sucrose solution at lower concentrations (e.g. 15% (w/w)) there are still negative consequences on physiology, and fecundity, demonstrating the bees are nutritionally stressed [59,64]. We hypothesized that exposure to flupyradifurone would impair bumblebee behaviour and that negative synergistic interactions would occur when bees were exposed to both the pesticide and lower sucrose concentrations.

## 2. Methods

### 2.1. General protocol

**Figure 1.**
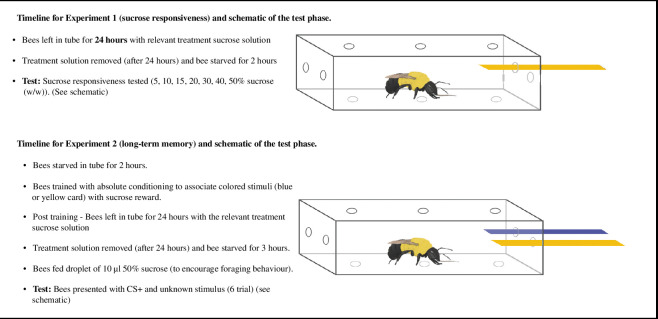
Timeline for experiment 1 (sucrose responsiveness) and experiment 2 (long-term memory retention). (Schematics demonstrate test phase of each experiment.)

To assess the impact of flupyradifurone and poor nutrition on both sucrose responsiveness (experiment 1) and long-term memory (experiment 2) we used a modified version of a free-moving proboscis extension reflex protocol (figure 1) [[38,65,66]]. Six bumblebee (*B. impatiens*) colonies (four for experiment 1 and two for experiment 2) were purchased (Koppert Biological Systems, Oxnard, CA, USA) and housed under laboratory conditions. Colonies were provided with an ad libitum supply of 50% (w/w) sucrose solution that bees could access from a wick in a plastic tube that was connected to the colony. Colonies were supplemented with honeybee-collected pollen (Koppert Biological Systems), which was poured directly into the colony boxes (1 tbsp every 2–3 days).

Bees were collected from colonies either with forceps (experiment 1) or with a bee vacuum (experiment 2) and placed directly into individual transparent plastic rectangular tubes (figure 1). In experiment 1 (sucrose responsiveness), we immediately inserted a glass tube containing an ad libitum supply of sucrose with the relevant pesticide and sucrose concentrations (see below for treatment details). In experiment 2 (long-term memory retention), bees were first trained to identify a rewarding colour stimulus (see below for details) before a glass tube containing the relevant treatment solution was added. In both experiments, bees were left for 24 h with the treatment concentrations before testing began. After 24 h, we removed the glass tubes, and recorded sucrose consumption with callipers [46]. From each sucrose consumption measurement, we subtracted the mean evaporation control from across the experiment (experiment 1 *n* = 25 and experiment 2 *n* = 40). After testing, all bees were placed in the freezer and we recorded their intertegular distance with callipers as a proxy for body size [67].

### 2.2. Pesticide and sucrose concentrations

Bees were provided with an ad libitum supply of sucrose from one of four treatments: (i) control, 50% (w/w) sucrose; (ii) control, 15% (w/w) sucrose; (iii) flupyradifurone (4 ppm) + 50% (w/w) sucrose; and (iv) flupyradifurone (4 ppm) + 15% (w/w) sucrose.

We based our pesticide exposure regime (4 ppm) on the concentration found in the nectar of honeybees foraging on winter-sown oilseed rape (*Brassica napus*) that had been treated with both seed and foliar applications of flupyradifurone (10 g ai/kg seed; 0.28 lbs/A foliar spray prior to sowing and 2 × 0.18 lbs ai/A during early and full flowering) [50]. The highest concentration found in the nectar of foraging honeybees (*Apis mellifera*) was 4.3 ppm and reached as high as 21 ppm in honeybee-collected pollen. The residue levels found in flowers ranged between 0.8 and 36 ppm. Given these high concentrations, our exposure regime (4 ppm) is probably an underestimate of a worst case but field-realistic scenario. We combined flupyradifurone (powder form, analytical standard from Chem Service, Pennsylvania, PA, USA) with water to create a stock solution that was added to the relevant sucrose solution (either 15% or 50% (w/w)) to create a 4 ppm solution. This solution was subset and frozen in Eppendorf’s and defrosted on the day of experiment to reduce degradation.

The low (15% (w/w)) and high (50% (w/w)) sucrose concentrations were chosen to represent a range of different nectar concentrations from bee-visited crops and wildflowers (reviewed in [68,69]). The nectar concentration of oilseed rape (which the pesticide concentration was based on) changes from approximately 30% (30 g 100 ml^-1^) at the start of flowering to approximately 10% (10 g/100 ml) at the end of flowering [70]. However, nectar concentrations of crops and wildflowers can be significantly lower or higher than this range. A recent review that surveyed flowers from 322 crops, wildflowers and weeds, found that concentrations of nectar ranged from 6.3% to 85% (w/w) [69]. We chose 50% as this concentration is (i) a high concentration of nectar found in bee-attractive crops, and (ii) because it is the concentration typically used in laboratory experiments that are incorporated into environmental risk assessments [71]. Fifteen per cent was chosen because it represents a low, but field-realistic concentration of nectar that is relevant to oilseed rape and other bee-attractive crops [69,70].

### 2.3. Experiment 1: sucrose responsiveness

We placed 144 bees in tubes. After 24 h, we removed the glass tubes containing the treatment solutions and starved bees for 2 h. Twenty-nine bees did not survive the exposure period (control 15% *n* = 9, control 50% *n* = 6, treatment 15% *n* = 8 and treatment 50% *n* = 6), 18 bees did not consume any of the sucrose solutions (control 15% *n* = 1, control 50% *n* = 4, treatment 15% *n* = 4 and treatment 50% *n* = 9), eight bees escaped, and the wick was pulled out of the glass tubes in three trials which meant we could not measure sucrose consumption. To test sucrose responsiveness, we dipped a strip of paper (dark yellow) in sucrose solution and used it to stimulate the bees’ antennae for 3 s (figure 1). In the first trial, we presented bees with just water. In each subsequent trial, we presented a sucrose solution in ascending concentrations from 5% (w/w) sucrose in the first trial up to 50% (w/w) in the final trial (5, 10, 15, 20, 30, 40 and 50% sucrose (w/w)). We tested an individual bee in quick succession [66], and in between each trial, we presented the bees with water [72]. We recorded a positive response when the bee extended her proboscis when stimulated with the sucrose solution. When a bee did extend her proboscis, she was allowed to feed on the sucrose for 3 s. We were unable to test four bees owing to aggressive behaviour, so our final sample sizes were 82 bees (control 15% *n* = 23, control 50% *n* = 21, treatment 15% *n* = 22 and treatment 50% *n* = 16).

### 2.4. Experiment 2: long-term memory

We placed individual bees (*n* =133) in tubes and starved them for 2 h. We trained each bee through absolute conditioning to associate a coloured stimulus with a sucrose solution (50% (w/w)). A coloured stimulus (either a dark blue or dark yellow coloured strip of card), was dipped in 50% (w/w) sucrose solution and used to stimulate the antenna of each bee for 3 s (CS+). As we used absolute conditioning to train the bees, only one colour was presented to each bee in the training phase. We repeated this process five times per bee, with an inter-trial interval of 10 min. If a bee did not extend her proboscis in response to the sucrose, we terminated their training (*n* = 10). The colour of the CS+ (either dark blue or yellow) was counterbalanced between bees. Immediately after training a glass tube containing an ad libitum supply of the relevant sucrose solution was inserted into the tube (see above).

We removed the treatment solutions after 24 h and starved the bees for 3 h. Sixteen bees had not consumed the sucrose (control 15% *n* = 3, 50% control *n* = 4, treatment 15% *n* = 2 and treatment 50% *n* = 7), two bees had escaped and 27 had died (control 15% *n* = 5, 50% control *n* = 9, treatment 15% *n* = 8 and treatment 50% *n* = 5). We then presented each bee with a droplet of 10 µl 50% (w/w) sucrose to stimulate foraging behaviour before testing began. To test long-term memory (defined as >24 h in honeybees [73]) we presented each bee with both stimuli (the CS+ and the unknown stimulus) for a maximum number of six trials (figure 1). Both strips had been dipped in water. We recorded a positive response if the bee approached and either antennated or extended their proboscis in response to the CS+. In order to maintain foraging motivation, the six trials were conducted in quick succession. The subsequent trial started immediately after a response from the bumblebee [66]. Most bees completed all six trials, but a subset (*n* = 10) completed less (mean number of trials = 5.57 ± s.d. 1.1). We therefore present the data as the proportion of correct choices made by each bee across the number of completed trials (see §2.5 for further details). Two bees were not motivated to approach either stimuli and were assumed to be unmotivated and removed from the experiment. Our final sample size was 76 bees (control 15% *n* = 20, control 50% *n* = 18, treatment 15% *n* = 22 and treatment 50% *n* = 16).

### 2.5. Statistical analysis

We used an information-theoretic model selection approach. We created a full model containing all the measured factors and created every possible subset of the full model and used Akaike information criterion for small sample size (AICc) values to determine the best model fit. In cases, when there was more than one model with an AICc value of less than 2, we used model averaging. For a list of all models used and all outputs produced, see the electronic supplementary material, tables S1 and S2. Each analysis was conducted in R (v.4.3.0). We used the packages *lme4* and *MuMIn* [74,75].

### 2.5.1. Experiment 1: sucrose responsiveness

To assess sucrose responsiveness, we analysed whether bees responded positively to each presented sucrose concentration [76]. A positive response was coded as ’1’ and an unresponsive bee as ‘0’. We used generalized linear mixed effect models with the colony and bee identity included as random factors. The full model contained pesticide (presence or absence), nutrition (15 or 50% (w/w) sucrose), bee size (intertegular distance), sucrose concentration tested (5, 10, 15, 20, 30, 40 and 50%), two-way interactions between: (i) pesticide and sugar concentration tested; (ii) nutrition and sucrose concentration tested; (iii) pesticide and nutrition; and (iv) a three-way interaction between pesticide, nutrition and sucrose concentration tested.

### 2.5.2. Experiment 2: long-term memory

To assess whether exposure to the treatment solutions influenced long-term memory, we analysed the proportion of correct approaches to the CS+. We used generalized linear mixed-effect models with a binomial distribution. The full model included pesticide, nutrition, bee size, CS+ colour and the interaction between pesticide and nutrition. Colony was also included as a random factor. As sensitivity analysis to ensure that extinction was not occurring, we conducted a follow-up analysis that analysed the interaction between test response and test number (1–6) and found no significant effect (test number: parameter estimate (PE) = −0.02, 95% confidence intervals (CI) = −0.08 to 0.06).

### 2.5.3. Sucrose consumption

For (i) sucrose solution (µl), and (ii) total sucrose (g) consumed in both experiments 1 and 2 (see above), we used linear mixed-effect models with pesticide, nutrition, bee size, the interaction between pesticide and nutrition all included in the full model, and colony included as a random factor.

## 3. Results

### 3.1. Experiment 1: sucrose responsiveness

**Figure 2.**
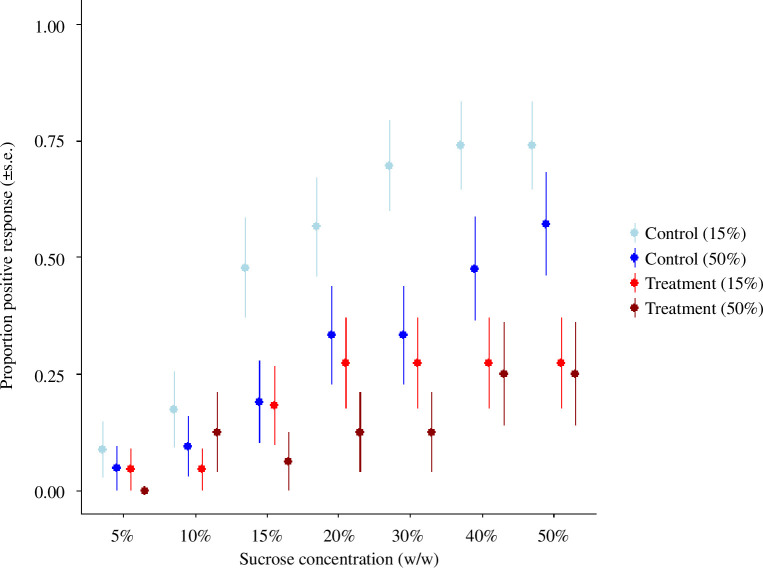
The proportion of bees (±s.e.) extending their proboscis in response to the sucrose concentration presented (control 15%, *n* = 23; control 50%, *n* = 21; treatment 15%, *n* = 22; treatment 50%, *n* = 16) (pesticide: PE = −4.68, CI = −7.59 to −1.77; nutrition: PE = 1.92, CI = −1.31 to 3.91; pesticide:nutrition: PE = −0.33, CI = −2.63 to 1.97).

We found that exposure to flupyradifurone impaired bumblebee sucrose responsiveness, but poor nutrition did not (figure 2; pesticide: PE = −4.68, CI = −7.59 to −1.77; nutrition: PE = 1.92, CI = −1.31 to 3.91). The interaction between the pesticide and nutrition was additive as opposed to synergistic or antagonistic (pesticide:nutrition: PE = −0.33, CI = −2.63 to 1.97). Unsurprisingly, the concentration of sucrose presented to the bees also influenced the likelihood of a positive response (sucrose concentration: PE = 0.12, CI = 0.09 to 0.16). Bee size did not influence sucrose responsiveness (electronic supplementary material, tables S1 and S2).

### 3.2. Experiment 2: long-term memory

**Figure 3.**
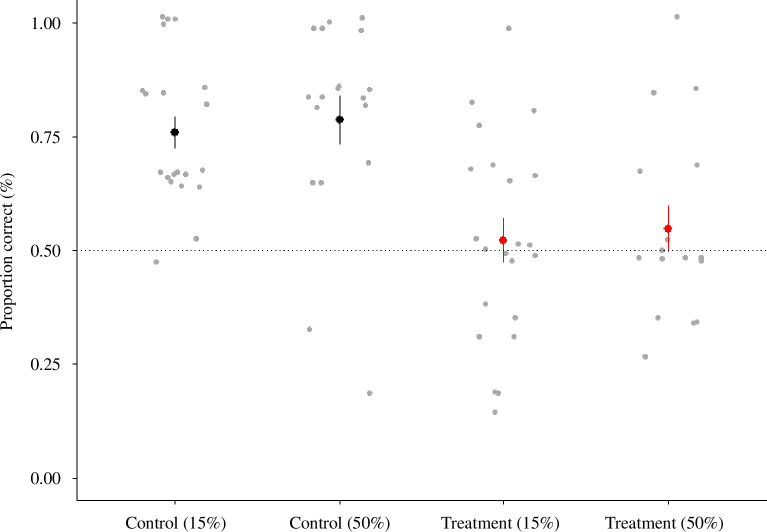
The mean (±s.e.) proportion of bees that chose the CS+. Grey dots are the results from individual bees (control 15%, *n* = 20; control 50%, *n* = 18; treatment 15%, *n* = 22; treatment 50%, *n* = 16) (pesticide: PE = −2.86, CI = −4.17 to −1.56; nutrition: PE = 0.08, CI = −0.56 to 0.72; pesticide:nutrition: AICc >2).

Flupyradifurone significantly impaired bumblebee long-term memory, but we found no impact of poor nutrition (figure 3; pesticide: PE = −2.86, CI = −4.17 to −1.56, nutrition: PE = 0.08, CI = −0.56 to 0.72). There was no significant interaction between pesticide and nutrition, suggesting an additive interaction (figure 3; electronic supplementary material, table S1, model with pesticide:nutrition AICc >2). Neither the colour of the CS+ nor the bee size influenced long-term memory (electronic supplementary material, tables S1 and S2).

### 3.3. Sucrose consumption

**Figure 4.**
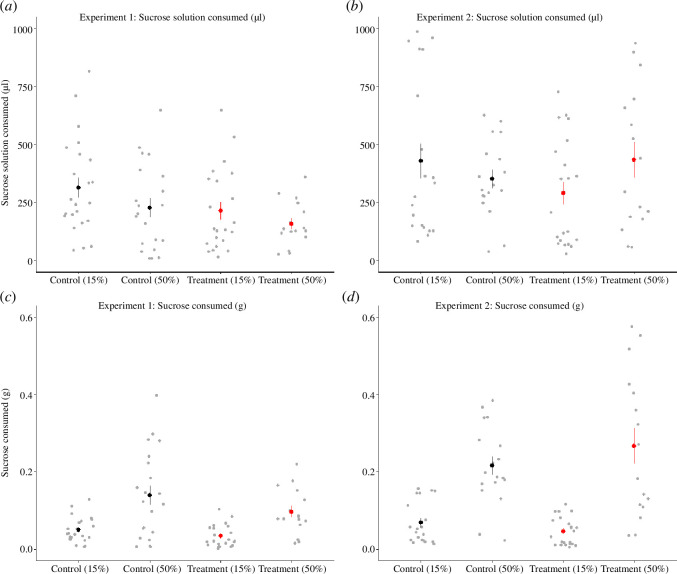
The mean (±s.e.) amount of sucrose solution (µl) (*a* and *b*) and sucrose (g) (*c* and *d*) consumed by individual bumblebees over 24 h before testing in (*a* and *c*) experiment 1 (sucrose sensitivity testing) and (*b* and *d*) experiment 2 (long-term memory testing). Grey dots indicate the result from individual bees. (*a* and *c*): control 15%, *n* = 23; control 50%, *n* = 21; treatment 15%, *n* = 22; treatment 50%, *n* = 16; (*b* and *d*): control 15%, *n* = 20; control 50%, *n* = 18; treatment 15%, *n* = 22, treatment 50%, *n* = 16. Experiments 1 and 2: sucrose solution (µl) consumed; any model containing pesticide, nutrition and pesticide:nutrition AICc >2. Experiment 1: sucrose (g) consumed, nutrition: PE = −0.09, CI = −0.11 to −0.06; models containing pesticide and nutrition: pesticide AICc >2. Experiment 2: sucrose (g) consumed, nutrition: PE = −0.17, CI = −0.21 to −0.14, models containing pesticide and nutrition:pesticide AICc >2.

We found no significant effect of pesticide or nutrition on the amount of sucrose solution (µl) consumed over 24 h in either experiment 1 or experiment 2 (figure 4*a,b*; electronic supplementary material, table S1: experiment 1, models with pesticide or poor nutrition, AICc >2; experiment 2, models with pesticide or poor nutrition, AICc >2). Larger bees consumed more sucrose across both experiments (electronic supplementary material, table S2). We did find significant differences in the total amount of sucrose (g) consumed between treatment groups in both experiments (figure 4*c*,*d*). Nutrition influenced the amount of sugar consumed by bees in both experiments (figure 4*c*; experiment 1, nutrition: PE = −0.09, CI = −0.11 to −0.06; figure 4*d*; experiment 2, nutrition: PE = −0.17, CI = −0.21 to −0.14), but there was no effect of pesticide, and no significant interaction between poor nutrition and pesticide (AICc >2; electronic supplementary material, tables S1 and S2).

## 4. Discussion

We found that exposure to flupyradifurone at field-realistic concentrations impaired bumblebee sucrose responsiveness and long-term memory. These negative effects occurred when bees were nutritionally poor or healthy, indicating that higher sugar intake did not buffer the negative consequences of pesticide exposure. Poor nutrition did result in sugar deficiency, but we found no evidence that this influenced bumblebee behaviour. The interactions between exposure to the pesticide and poor nutrition were additive (rather than synergistic or antagonistic) but were of little consequence as sucrose concentration had no observable impact on bumblebee behaviour. The negative impact of flupyradifurone exposure on both sucrose responsiveness and long-term memory is similar to those observed with neonicotinoids [18,33]. This suggests that, at least in relation to bumblebee behaviour, banning one group of pesticides will not protect pollinators from the negative impacts of pesticides without simultaneous improvements to agrochemical risk assessment [55,77].

Flupyradifurone has been termed ‘bee safe’ by regulators, and so can be used on bee-visited crops while they are flowering [50]. Despite this, previous studies have found that flupyradifurone can impair bee behaviour [55]. For example, exposure to flupyradifurone can impair olfactory learning in Asian honeybees (*Apis cerana*) at field-realistic concentrations [51], and acute exposure to flupyradifurone can also impair bumblebee olfactory/colour learning and memory [38]. Here, we found that bumblebees exposed to flupyradifurone responded positively to 50% sucrose in 26.3% of trials compared with 65.9% in control groups. We also found that in a long-term memory test, bumblebees exposed to the flupyradifurone responded correctly in 53.2% of trials, compared with 77.1% in control bees. In contrast to this, exposure to field-realistic concentrations of flupyradifurone does not appear to influence sucrose responsiveness and learning in western honeybees (*A. mellifera*) [78]. It is unclear why there are species-level differences in how flupyradifurone influences bee behaviour, but honeybees (*A. mellifera*) are often more robust to pesticide exposure than wild bees [10]. Our data add to a growing body of evidence indicating that honeybees are not a representative model species for pesticide risk assessments [77,79].

Pollen consumption and composition can influence bee behaviour [80–82], but less is known about the behavioural and cognitive consequences of lower carbohydrate intake by bumblebees. Here, we found no evidence that sugar deficiency impairs bumblebee sucrose responsiveness or long-term memory. In one of the few studies to assess the impact of sugar deficiency on insect learning, wasps (*Venturia canescens*) fed honey performed better in an olfactory learning task compared with those fed sucrose solution (20% (w/w)) [83]. Honey, however, contains more nutrients (e.g. amino acids and minerals) than just sucrose which could drive the observed differences in learning [83]. While we found no evidence to suggest that sugar deficiency influences the behaviour of adult bumblebees over 24 h, the impact of long-term low carbohydrate intake on pollinator behaviour is still relatively unexplored and warrants further research.

Poor nutrition can make bees more vulnerable to pesticide exposure, but most previous research has focused on the interactions between pesticides and pollen intake, or floral diversity [24–27,84–86]. We found that flupyradifurone impaired sucrose responsiveness and long-term memory when bumblebees were nutritionally stressed and healthy, indicating that higher carbohydrate intake did not buffer the negative consequences of pesticide exposure [30]. This is not surprising as negative effects of pesticides on bees regularly occur even when bees are fed high sucrose concentrations [41,45,87]. Indeed, 50% (w/w) sucrose solution is used as a standard concentration in pesticide risk assessment [62]. Contrary to what we expected, we found no significant interaction between nutrition and pesticides in relation to bee behaviour. The sub-lethal effects of pesticides are correlated with dose, and we had predicted that bees fed 15% (w/w) sucrose would consume more than those fed 50% (w/w), which would have increased the dose of pesticide they consumed [13]. However, we found no significant differences in the amount of sucrose solution consumed between bees from different treatment groups. This was probably caused by bees not being able to store sucrose in their nest, which limited consumption. Despite this, bees in the 15% (w/w) groups consumed less total sugar, which can impair bumblebee fitness [13,64]. Future studies that chronically expose whole colonies to a combination of pesticides and poor nutrition are required to better understand these key interactions. However, given that the interactions between poor nutrition and pesticide exposure can have significant negative impacts on wild bee fitness [23–27,84–86,88], incorporating these key interactions into pesticide risk assessment should be a priority for policymakers interested in creating a more holistic, and field-realistic regulatory process [55,77].

Our results demonstrate that despite being labelled as ‘bee safe’, flupyradifurone had a significant negative effect on bumblebee sucrose responsiveness and long-term memory. These data add to a growing body of evidence demonstrating that novel insecticides such as flupyradifurone, which could replace neonicotinoid use over a large geographical area, have significant negative consequences on pollinators [14,55,89–92]. In the short term, regulators should restrict flupyradifurone use to non-flowering crops, while a re-assessment of its potential risk to pollinators can be re-evaluated. Ultimately, our results, and those that came before [14,55,89–92], have demonstrated that pesticide risk assessments globally are failing to protect bees from the unintended consequences of pesticide use. Consequently, banning or restricting the use of certain agrochemicals (e.g. neonicotinoids in the European Union and United Kingdom) will not protect bees from the negative impacts of pesticide use without concurrent changes to environmental risk assessment [55,77,93–95].

## Data Availability

All data will be available online via OSF [96]. Electronic supplementary material is also available online [97].
